# Fiber enrichment is not superior to dietary monitoring in MASLD: A dual-center, double-blind, placebo-controlled trial

**DOI:** 10.1016/j.isci.2025.114019

**Published:** 2025-11-11

**Authors:** Annette Brandt, Timur Yergaliyev, Emina Halibasic, Aline Cyba, Julius W. Jaeger, Rongpeng Gong, Angélica Hernández-Arriaga, Carolin Victoria Schneider, Wilhelm Sjöland, Antonio Molinaro, Michael Trauner, Christian Trautwein, Amélia Camarinha-Silva, Ina Bergheim, Kai Markus Schneider

**Affiliations:** 1 Department of Nutritional Sciences, Molecular Nutritional Science, University of Vienna, Vienna, Austria; 2 Livestock Microbial Ecology Department, Institute of Animal Science, University of Hohenheim, Stuttgart, Germany; 3 Division of Gastroenterology and Hepatology, Department of Medicine III, Medical University of Vienna, Vienna, Austria; 4 Department of Internal Medicine III, Gastroenterology, Metabolic Diseases and Intensive Care, University Hospital RWTH Aachen, Aachen, Germany; 5 Department of Medicine I, Department of Gastroenterology and Hepatology, Faculty of Medicine and University Hospital Carl Gustav Carus, TUD Dresden University of Technology, Dresden, Germany; 6 Center for Regenerative Therapies Dresden (CRTD), Technische Universität (TU) Dresden, Dresden, Germany; 7 Else Kroener Fresenius Center for Digital Health, Faculty of Medicine and University Hospital Carl Gustav Carus, TUD Dresden University of Technology, Dresden, Germany; 8 The Institute for Translational Medicine and Therapeutics, the Perelman School of Medicine, University of Pennsylvania, Philadelphia, PA, USA; 9 Department of Molecular and Clinical Medicine, Wallenberg Laboratory, University of Gothenburg, Gothenburg, Sweden; 10 The Krantz Family Center for Cancer Research, The Massachusetts General Hospital Cancer Center, Harvard Medical School, Boston, MA, USA; 11 Department of Molecular and Clinical Medicine, Wallenberg Laboratory, University of Gothenburg, Gothenburg, Sweden; 12 Leibniz Research Centre for Working Environment and Human Factors at the TU Dortmund (IfADo), Dortmund, Germany; 13 Leberzentrum Stuttgart, Klinik für Gastroenterologie, Gastrointestinale Onkologie, Hepatologie und Infektiologie, Klinikum Stuttgart, Stuttgart, Germany; 14 ERN Rare Liver Center Sahlgrenska University Hospital, Gothenburg, Sweden

**Keywords:** clinical nutrition, human metabolism

## Abstract

Dietary fiber enrichment may modulate intestinal microbiota and positively impact metabolic dysfunction-associated steatotic liver disease (MASLD). This randomized, double-blind, placebo-controlled dual-center study evaluated the effects of dietary fiber (oat bran and spelt bran) on MASLD. After a 3-week Run-in phase during which dietary intake was assessed, 48 patients (CAP >280 dB, no fibrosis) were assigned to oat bran (4.5 g oat β-glucan, total fiber 11.7 g/day), spelt bran (11.7 g fiber/day), or placebo (2.1 g fiber/day) for 12 weeks. During the Run-in phase, dietary assessment alone significantly decreased BMI and liver enzymes (ALT, AST, γ-GT) while increasing microbiota diversity. Improvements were maintained in all three intervention groups. However, no significant changes were observed in hepatic steatosis (CAP), overall microbiota composition, and serum bile acid profiles. Dietary assessment alone improved MASLD biomarkers, with the fiber supplementation offering no additional benefit. This highlights the importance of dietary counseling in MASLD management. (clinical trials: NCT03897218).

## Highlights

• Dietary assessment alone lowers BMI and liver enzymes in patients with MASLD
• Fiber supplementation showed no adverse effects
• Enriching the diet of patients with MASLD had no additional effect on BMI or liver enzymes
• Fiber supplementation had no effects on microbiota or bile acid composition

## Graphical abstract

**Figure:**
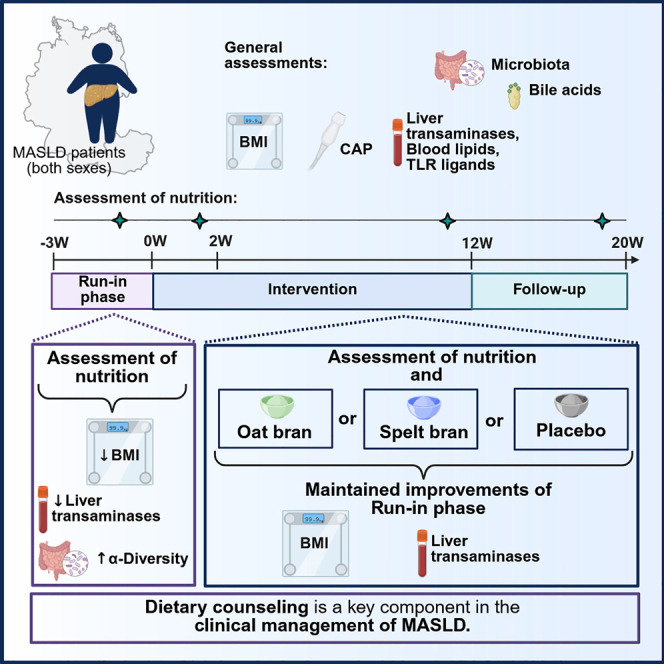

## Introduction

Metabolic dysfunction-associated steatotic liver disease (MASLD) affects approximately 25–30% of the global population, making it the most common liver disease worldwide.^1^^,^^2^ MASLD encompasses a spectrum ranging from simple steatosis, characterized by triglyceride accumulation in hepatocytes, to metabolic dysfunction-associated steatohepatitis (MASH), fibrosis (with an annual progression rate of ∼40.76% in MASH), cirrhosis, and hepatocellular carcinoma.^3^ Despite significant research, the pathogenesis of MASLD remains incompletely understood, and effective preventive and therapeutic strategies beyond general lifestyle modifications are still limited, particularly for patients at early disease stages.

Besides general overnutrition, specific dietary patterns have been proposed to contribute to MASLD risk.^4^ Diets high in sugars (e.g., fructose, sucrose), saturated fats, and cholesterol, combined with low fiber consumption, are frequently associated with MASLD development.^5^^,^^6^ Such dietary patterns have been suggested to alter the gut microbiota composition and intestinal barrier function. The imbalance in gut microbiota may increase intestinal permeability, commonly referred to as “leaky gut,” allowing the translocation of microbial products such as lipopolysaccharides, into the portal circulation.^7^^,^^8^^,^^9^ This process links diet-induced microbiota changes to liver disease progression. Patients with MASLD often exhibit increased intestinal permeability, endotoxemia, and Toll-like receptor 4 (TLR4) activation.^10^ The translocation of bacterial endotoxins activates Kupffer cells in the liver, promoting inflammation and contributing to disease progression.^8^

The liver also influences gut physiology through bile acids and enterohepatic circulation.^11^ Patients with MASLD exhibit significant alterations in bile acid composition.^12^ Bile acids and microbial bile acid metabolites play crucial roles in gut-liver crosstalk, acting as signaling molecules that regulate metabolic pathways and inflammatory responses through bile acid receptors such as TGR5 and FXR.

Increasing dietary fiber intake through introducing whole grains, resistant starch or wheat bran into the diet has been suggested to improve health markers and change intestinal microbiota composition.^13^ Due to the complexity and diversity of dietary fibers, it seems that different types of dietary fibers, such as soluble or insoluble, viscous or non-viscous, but also fibers of various origins differ in their potential health benefits.^14^ Oat β-glucans, soluble, highly viscous and gel-forming fibers found in oats, known for lowering LDL and total cholesterol without affecting HDL levels, may mitigate MASLD, by modulating gut microbiota and enhancing short-chain fatty acid (SCFA) production.^15^^,^^16^ Despite the potential of pro-, pre-, or symbiotic in MASLD, effective therapies remain underdeveloped. A daily intake of 3 g oat β-glucans has been shown to reduce LDL and total cholesterol, leading to health claims endorsed by the European Food Safety Authority (EFSA).^17^ A small study showed that 3 g/day oat β-glucans reduced serum ALT and AST levels in overweight individuals with altered liver function.^18^ Moreover, in a pilot study, we found that the weeklong intake of oat bran (4.5g β-glucans, total dietary fiber: 11.7g) was related to a reduction in ALT and AST levels as well as bacterial endotoxin levels in healthy women, while similar effects were not found after the intake of spelt bran.^19^ Also, our previous research demonstrated a protective effect against MASLD progression toward fibrosis in mice.^20^ The beneficial effects of oat β-glucans may result from gut microbiota modulation, and increased SCFA production, as β-glucans have been shown to promote beneficial bacteria and shift gut microbial metabolism in animal models.^20^ On the other hand, insoluble fibers found in spelt bran are characterized by low fermentation rates and the ability to increase stool moisture and bulk.^21^ They may also activate cellular and molecular anti-inflammatory mechanisms.^22^ However, whether fortifying the diet of patients with MASLD with oat bran, being rich in soluble fiber, improves liver function in human MASLD and the underlying mechanisms remain unclear.

Here, we conducted a randomized, double blind, placebo-controlled study to evaluate the effects of nutritional monitoring and enrichment of the diet with different fibers, e.g., oat bran, which is rich in soluble fiber, and spelt bran which is rich in insoluble fiber, compared to a placebo with low fiber content in patients with MASLD.

## Results

### Intervention implementation and participant compliance

**Figure 1:**
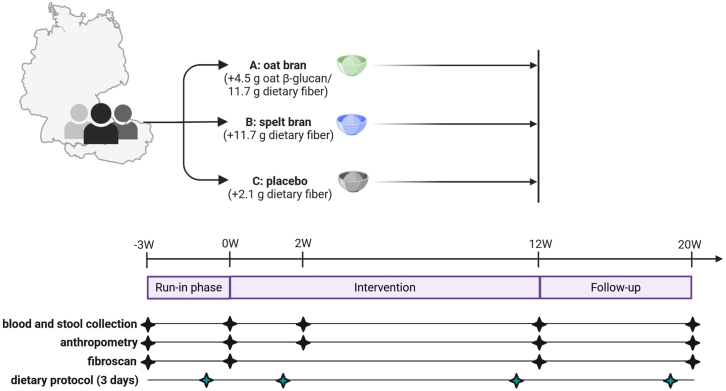
Study design

From April 2019 to October 2021, 79 participants were assessed for eligibility from two research centers (Aachen, Germany, and Vienna, Austria). Forty-eight participants were randomized to receive either oat bran (Group A, *n* = 16), spelt bran (Group B, *n* = 17), or a placebo (Group C, *n* = 15) (Figure 1). Thirty-four participants completed the entire study protocol, provided all stool samples, and were included in the final analysis (Group A, *n* = 12; Group B, *n* = 11; Group C, *n* = 11). Overall, all flake mixes were well tolerated, and dropout rates were similar between the groups. Dropouts during the study were due to diarrhea (*n* = 1), stomach pain (*n* = 1), knee surgery (*n* = 1), and personal reasons (*n* = 4), while one participant had to be excluded due to stress eating behavior during exams. All participants completed scheduled sample collections, though six samples were excluded from analysis due to quality issues (see Figure S1 CONSORT Flow Diagram).

### Impact of nutritional assessment on steatotic liver disease during the Run-in phase

**Figure 2:**
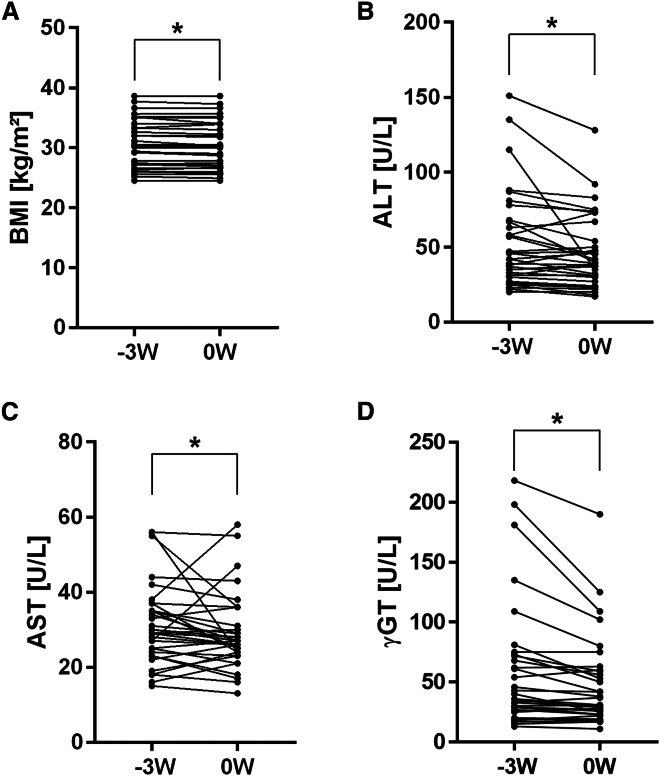
Body weight and serum markers of liver damage at the Run-in phase (A) BMI (paired *t* test, ∗*p* < 0.05, *n* = 34), (B) alanine aminotransferase (ALT) activity (Wilcoxon test, ∗*p* < 0.01, *n* = 34), (C) aspartate aminotransferase (AST) activity (Wilcoxon test, ∗*p* < 0.05, *n* = 34), and (D) ɣ glutamyl transferase (ɣ-GT) (Wilcoxon test, ∗*p* < 0.001, *n* = 34) in serum of patients at timepoint -3W and 0W. Single data points are presented in each graph.

To assess the impact of the introduction of the different fiber rich flake mixes and the placebo on dietary habits and nutrient intake, all participants were requested to document their food and beverage intake, filling in a 3-day weighing protocol. While not being advised to change their diet except for the intake of oat-containing foods during the 3-week Run-in period (-3W to 0W), mean serum liver enzymes of participants, including ALT, AST, and γ-GT, improved significantly. Moreover, the BMI of patients was also significantly reduced (*p* < 0.05) (Figures 2A–2D), while neither blood lipids, fasting blood glucose, or CAP differed between -3W and 0W (see Table 1).

**Table 1:** Effect of Run-in phase on markers of liver damage and blood lipids

	-3W	0W	*p*-value
N	34		–
Sex [m/f]	17/17		–
Age [years]	48.8 ± 13 [44.3, 53.4]		–
Body weight change from 0W to -3W [kg]	-0.33 ± 1.2 [-0.76, 0.10]		–
waist circumference [cm]	102 ± 11 [98.5, 106]	102 ± 11 [98.2, 106]	0.539
CAP dB/m	322 ± 42 [308, 337]	320 ± 39 [306, 334]	0.745
Liver Stiffness, kPa	5.3 ± 1.2 [4.9, 5.7]	5.5 ± 1.6 [4.9, 6.1]	0.493
Fasting glucose [mg/dL]	100 ± 23^a^ [91.9, 109]	97.5 ± 17 [91.6, 104]	0.241
Cholesterol [mg/dL]	198 ± 54^a^ [179, 217]	196 ± 48 [180, 213]	0.634
HDL-cholesterol [mg/dL]	47.4 ± 16^b^ [41.1, 53.8]	48.8 ± 15^c^ [43.1, 54.5]	0.858
LDL-cholesterol [mg/dL]	123 ± 45^b^ [105, 141]	123 ± 38^c^ [109, 137]	0.412
Triglycerides [mg/dL]	168 ± 87^a^ [137, 198]	193 ± 154 [139, 246]	0.270

Mean ± SD as well as 95% confidence interval [lower limit, upper limit], paired *t-test* or Wilcoxon test.

a one value is missing.

b 7 values missing.

c 4 values missing.

**Figure 3:**
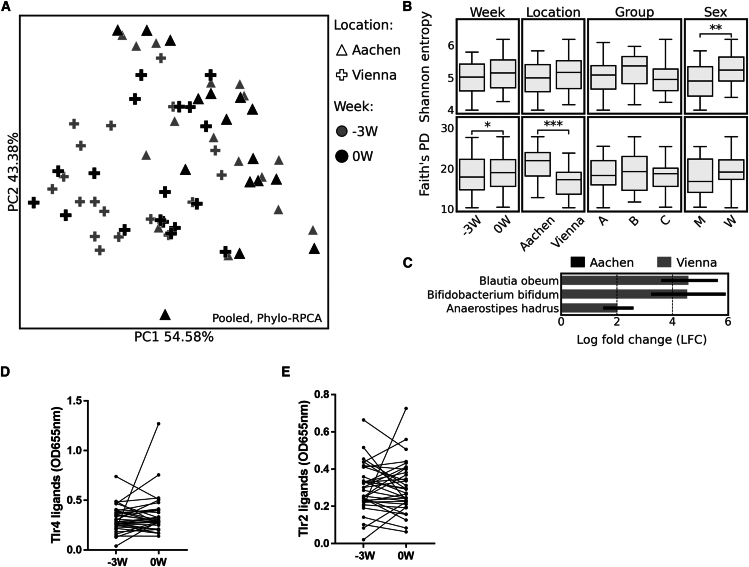
Microbiome assessment and TLR2 and 4 ligands at the Run-in phase (A) PCoA based on phylo-RPCA distances. Locations are differentiated by markers and weeks by color. (B) Shannon entropy and Faith phylogenetic diversity boxplots by week, location, group, and sex. The line inside the box represents the median, while the whiskers represent the lowest and highest values (excluding outliers) and interquartile range (IQR). Independent samples were tested with the Kruskal-Wallis test, and dependent samples were tested with the Wilcoxon test. Significant *p*-values are indicated with asterisks (∗ - *p* < 0.05; ∗∗ - *p* < 0.01; ∗∗∗ - *p* < 0.001). (C) ANCOM-BC differential abundance test between locations. Colors indicate where a given taxon was found to be more abundant. Log fold changes plotted only for taxons with adjusted *p*-values less than 0.05. (D) Toll-like receptor 4 (TLR4) (Wilcoxon test, *n* = 34) and (E) TLR2 ligands (Wilcoxon test, *n* = 34) in the serum of participants at timepoint -3W and 0W. Single data points are presented in graph D and E.

Using shotgun metagenomics sequencing baseline microbiome analysis revealed that while study location (Aachen vs. Vienna) was the primary factor influencing microbiota composition and phylogenetic diversity (PERMANOVA test, PCoA analysis, *p* < 0.01; Figures 3A and 3B; Table S1) and Shannon diversity index showed significant differences between sexes (*p* < 0.01) with higher values among female participants (Figure 3B) these changes in liver enzymes and BMI were related with systematic changes in microbiota composition. However, neither concentration of Toll-like receptor (TLR) 2 and 4 ligands in serum differs between -3W and 0W (Figures 3D and 3E).

### Effect of the intake of oat bran, spelt bran, or placebo on markers of steatotic liver disease

While BMI and serum markers of liver disease were all significantly lower after the 3 weeks Run-in phase, clinical parameters at week 0 showed no significant differences between groups, confirming effective randomization (Table 2). Still, cholesterol levels in serum were significantly higher in the spelt bran group at week 0 compared to the oat bran group. Only *n* = 7 participants in the oat bran and spelt bran group, respectively, and *n* = 8 in the placebo group provided dietary protocols for all four timepoints (0W, 2W, 12W, and 20W). Still, data suggests good compliance. Indeed, although the diets were not significantly impacting the overall dietary fiber intake according to the linear mixed effects (LME) test, the spelt bran significantly interacted with time, indicating that patients increased their daily fiber intake during the intervention period (*p* = 0.009, Table 3; Table S2). Interestingly, the daily dietary fiber intake was significantly reduced back to the initial quantity during Follow-up in both groups receiving the fiber rich flake mixes (week: oat bran: *p* < 0.001, week: spelt bran: *p* < 0.003) (Table 3; Table S2). Average kcal intake and the composition of macronutrients did not change in any of the groups during the intervention (Table 3; Table S2).

**Table 2:** Baseline characteristics 0 W

	oat bran	spelt bran	placebo
N	12	11	11
Sex [m/f]	6/6	6/5	5/6
Age [years]	51.0 ± 15	50.2 ± 14	49.7 ± 9.6
BMI [kg/m^2^]	31.0 ± 3.3	30.2 ± 4.2	30.4 ± 4.3
CAP dB/m	317 ± 42	325 ± 40	322 ± 35
ALT [U/L]	42.0 ± 23	45.3 ± 29	48.9 ± 22
AST [U/L]	26.3 ± 10	31.4 ± 11	31.6 ± 12
ɣ-GT [U/L]	41.4 ± 29	58.9 ± 50	39.1 ± 28
Fasting glucose [mg/dL]	97.9 ± 10^a^	98.6 ± 19	103 ± 27
Cholesterol [mg/dL]	173 ± 28	213 ± 46^c^	207 ± 37
HDL-cholesterol [mg/dL]	48.3 ± 14^a^	46.2 ± 19^a^	47.3 ± 13^a^
LDL-cholesterol [mg/dL]	103 ± 21^a^	133 ± 43^a^	131 ± 42^b^
Triglycerides [mg/dL]	141 ± 70	270 ± 238	172 ± 82

Mean ± SD, one-way ANOVA or Kruskal-Wallis test; CAP, controlled attenuation parameter; ALT, alanine aminotransferase; AST, aspartate aminotransferase; ɣ-GT, ɣ-glutamyl transferase.

a one value is missing.

b two values are missing.

c *p* < 0.05 compared to oat bran.

**Table 3:** Nutritional intake during dietary fiber and placebo intervention

nutrients/day	oat bran				spelt bran				placebo			
	0W	2W	12W	20W	0W	2W	12W	20W	0W	2W	12W	20W
kcal	2186 ± 426	2189 ± 593	2044 ± 463	1989 ± 873	1615 ± 404	1877 ± 369	1871 ± 427	1770 ± 419	2075 ± 744	2226 ± 669	2040 ± 642	1948 ± 616
g protein	93.5 ± 20	93.8 ± 21	82.1 ± 19	83.9 ± 32	68.1 ± 14	74.6 ± 16	78.9 ± 18	70.5 ± 18	80.5 ± 38	93.4 ± 25	86.3 ± 20	72.4 ± 24
g fat	99.3 ± 28	96.8 ± 32	85.3 ± 20	95.7 ± 33	77.4 ± 22	88.1 ± 22	83.3 ± 26	87.0 ± 20	88.6 ± 50	94.3 ± 33	87.3 ± 29	92.2 ± 37
g carbohydrates	210 ± 79	217 ± 75	224 ± 79	194 ± 125	155 ± 41	185 ± 42	189 ± 42	166 ± 56	197 ± 96	240 ± 73	218 ± 82	200 ± 50
g dietary fiber	18.7 ± 2.9	26.5 ± 4.8	25.0 ± 7.1	16.5 ± 7.6	16.5 ± 6.4	25.1 ± 8.8	26.9 ± 12	19.4 ± 9.2	17.6 ± 6.9	17.6 ± 5.9	16.0 ± 4.3	15.5 ± 3.6
HEI	71.1 ± 8.1	72.4 ± 7.7	69.8 ± 8.8	59.6 ± 13	72.3 ± 9.0	73.5 ± 8.9	69.5 ± 14	68.0 ± 13	69.9 ± 9.8	68.8 ± 6.5	66.2 ± 7.5	66.8 ± 12

Mean ± SD, oat bran *n* = 7, spelt bran *n* = 7, placebo *n* = 8, Linear mixed effects (LME) analyses were performed for intervention (weeks 0–12) and follow-up (weeks 12–20) stages with the placebo group as reference. Detailed LME analysis see Table S2.; Abbreviations: HEI – healthy eating index.

The placebo group continuously consumed ∼16-18 g of dietary fiber/day throughout the whole study. Further suggesting that dietary intake was not fundamentally changed by the introduction of the flake mixes, the healthy eating index (HEI) also remained similar during the study within each treatment group and did not differ between treatments (Table 3; Table S2).

### Liver function and controlled attenuation parameter changes during intervention and follow-up

**Figure 4:**
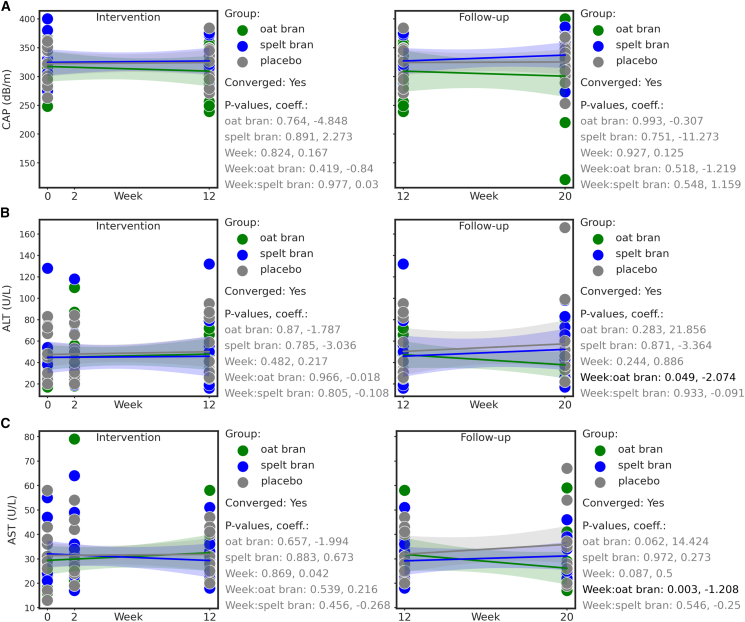
CAP and serum marker of liver damage during dietary fiber and placebo intervention Linear mixed effects analyses were performed for intervention (weeks 0–12) and follow-up (weeks 12–20) stages with the placebo group as reference. (A) CAP (oat bran *n* = 12, spelt bran *n* = 11, placebo *n* = 11), (B) alanine aminotransferase (ALT) activity (oat bran *n* = 12 expect for 2W *n* = 11, spelt bran *n* = 11 expect for 2W *n* = 10, placebo *n* = 11)) and (C) aspartate aminotransferase (AST) activity (oat bran *n* = 12 expect for 2W *n* = 11, spelt bran *n* = 11, placebo *n* = 11), in serum of patients at timepoint 0W to 20W. Bands represent 95% confidence intervals. *p*-values and coefficients are plotted on the right side of the subplots.

Following the run-in period, controlled attenuation parameter (CAP) and liver enzymes were monitored at different time points during the intervention and Follow-up phase. CAP values remained relatively stable across all groups (participants oat bran *n* = 12, spelt bran *n* = 11, placebo *n* = 11) with no significant differences detected by the linear mixed effects test (Figure 4A), which is in line with unchanged BMI during the study (Table 4; Table S3).

**Table 4:** BMI, markers of liver damage and total cholesterol as well as triglycerides during dietary fiber and placebo intervention

parameters	oat bran				spelt bran				placebo			
	0W	2W	12W	20W	0W	2W	12W	20W	0W	2W	12W	20W
BMI (kg/m^2^)	31.0 ± 3.3	31.0 ± 3.2	31.1 ± 3.2	30.7 ± 3.0	30.2 ± 4.2	29.4 ± 3.2	30.0 ± 4.5	30.2 ± 4.3	30.4 ± 4.3	30.3 ± 4.4	30.6 ± 4.3	30.4 ± 4.3
LiverStiffness (kPa)	4.9 ± 1.8	–	5.4 ± 1.7	6.1 ± 2.5	5.4 ± 1.0	–	5.1 ± 1.3	5.7 ± 0.8	5.7 ± 1.9	–	5.5 ± 1.5	5.4 ± 1.8
Cholesterol (mg/dL)	173 ± 28	169 ± 26	173 ± 33	172 ± 44	213 ± 46	207 ± 53	213 ± 38	207 ± 38	207 ± 37	189 ± 45	218 ± 64	207 ± 36
ɣ-GT (U/L)	41.4 ± 29	50.1 ± 51	52.6 ± 71	36.7 ± 26	58.9 ± 50	62.5 ± 53	60.3 ± 38	64.6 ± 40	39.1 ± 28	34.5 ± 16	38.8 ± 21	38.9 ± 16
Triglycerides (mg/dL)	141 ± 70	139 ± 63	169 ± 116	140 ± 73	270 ± 238	192 ± 85	256 ± 136	170 ± 76	172 ± 82	156 ± 45	190 ± 160	233 ± 210

Mean ± SD. Linear mixed effects (LME) analyses were performed for intervention (weeks 0–12) and follow-up (weeks 12–20) stages with the placebo group as reference. Detailed LME analysis, see Table S3. BMI (oat bran *n* = 12, spelt bran *n* = 11, except for 2W *n* = 10, placebo *n* = 11), liver stiffness (oat bran *n* = 12, spelt bran *n* = 11, placebo *n* = 11), cholesterol (oat bran *n* = 12, except for 2W and 12W *n* = 11, spelt bran *n* = 11, placebo *n* = 11, except for 2W *n* = 9), ɣ-GT (oat bran *n* = 12, except for 2W *n* = 11, spelt bran *n* = 11, placebo *n* = 11), triglycerides (oat bran *n* = 12, except for 2W *n* = 11, spelt bran *n* = 11, placebo *n* = 11, except for 2W *n* = 9).

Somewhat in line with the findings for CAP, ALT, and AST activities in serum remained similar throughout the intervention (Figures 4A–4C). Interestingly, both ALT and AST activity significantly decreased during follow-up (LME analysis, ALT: week: oat bran: *p* = 0.049, AST: week: oat bran: *p* = 0.0003) (Figures 4B and 4C).

Neither the intake of spelt bran nor of oat bran led to differences in cholesterol or triglyceride serum levels compared to the placebo group (Table 4; Table S3) during the intervention. However, triglyceride serum levels were affected in the spelt bran group, with a significant decline during the follow-up stage (Table 4; Table S3).

### Effect of oat bran and spelt bran on serum bile acid, gut microbiota composition, and TLR2 and 4 serum levels

**Figure 5:**
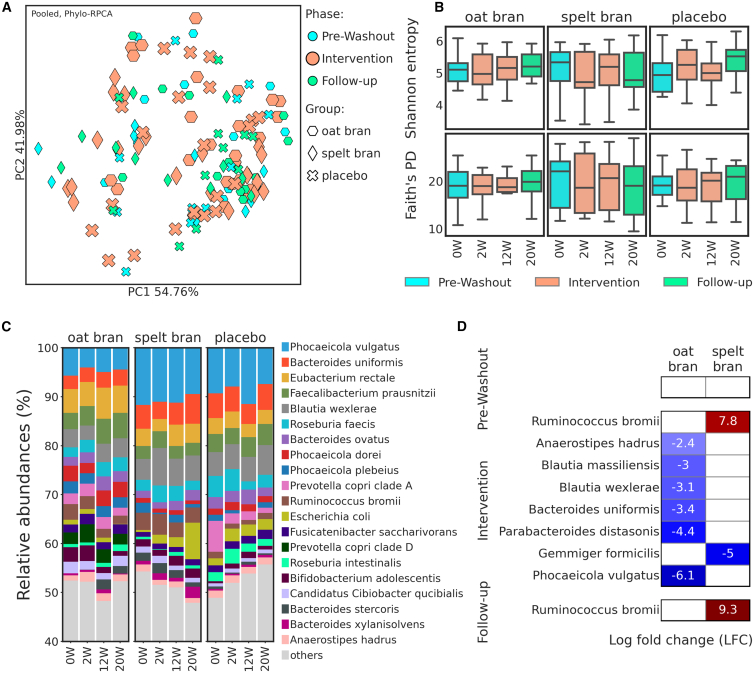
Microbiome assessment (A) PCoA plots based on phylo-RPCA distances. Groups are differentiated by markers and phases by color and marker size. (B) Shannon entropy and faith phylogenetic diversity boxplots by week for each group. The line inside the box represents the median, while the whiskers represent the lowest and highest values (excluding outliers) and interquartile range (IQR). (C) Taxonomy barplot at species level (top 20). Samples were averaged by week for each group. (D) ANCOM-BC differential abundance test between the placebo group and groups with intervention (oat and spelt bran). Log fold changes (LFC) were plotted only for taxons with adjusted p-values less than 0.05. Positive LFC values have a red background and indicate that a given taxon was more abundant in the intervention group compared to the placebo. In contrast, negative LFC values have a blue background and were more abundant in the placebo group.

Serum bile acid levels remained unchanged across all groups (Table S4). Microbial β-diversity analysis did not show a significant separation between intervention groups (oat bran and spelt bran) and placebo during both intervention (0-12 W) and Follow-up (12-20 W) periods. The only difference was found between the oat bran and spelt bran group during the intervention period (PERMANOVA, P-adj = 0.042; Figure 5A; Table S1). At the same time, α-diversity metrics showed no significant differences between groups.

ANCOM-BC differential abundance testing revealed microbial signatures between intervention groups and placebo. The oat bran group showed significantly reduced abundance of multiple species during the intervention period compared to placebo, including *Phocaeicola vulgatus* (LFC = −6.1), *Parabacteroides distasonis* (LFC = −4.4*), Bacteroides uniformis* (LFC = −3.4), *Blautia wexlerae* (LFC = −3.1), *Blautia massiliensis* (LFC = −3.0), and *Anaerostipes hadrus* (LFC = −2.4) (Figure 5D). In contrast, the spelt bran group showed limited impact on microbiota composition, with *Ruminococcus bromii* remaining consistently enriched compared to placebo both in Intervention (LFC = 7.8) and Follow-up periods (LFC = 9.3). Another intervention-related change observed in the spelt bran group was a reduction in *Gemmiger formicilis* (LFC = −5.0) compared to placebo, which was not observed during the Follow-up phase. All reported differences were statistically significant with adjusted *p*-values <0.05. Although differentially abundant species were detected between the placebo and experimental groups, no effect of the diet was observed on the functional profiles of the samples.

**Figure 6:**
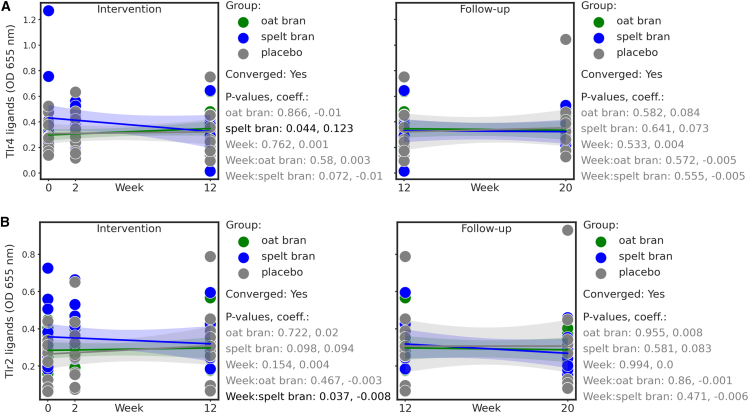
TLR2 and 4 ligands during dietary fiber and placebo intervention Linear mixed effects analyses were performed for intervention (weeks 0–12) and follow-up (weeks 12–20) stages with the placebo group as reference. (A) Toll-like receptor 4 (TLR4, oat bran *n* = 12 expect for 20W *n* = 11, spelt bran *n* = 11 expect for 20W *n* = 10, placebo *n* = 11) and (B) TLR2 ligands (oat bran *n* = 12 expect for 20W *n* = 11, spelt bran *n* = 11 expect for 20W *n* = 10, placebo *n* = 11) in serum of patients at timepoint 0W to 20W. Bands represent 95% confidence intervals. *p*-values and coefficients are plotted on the right side of the subplots.

The concentration of TLR4 ligands in serum, thought to be indicative of intestinal barrier function, was shown to be higher in the spelt bran group compared to the placebo during the intervention period, but gradually decreased between week 0 and week 12 (week: spelt bran, *p* = 0.072) (Figure 6A). At the same time, TLR2 ligands demonstrated a significant decrease from the start to the end of the intervention (week: spelt bran, *p* = 0.037) (Figure 6B). However, it should be noted that both TLR4 and TLR2 had higher levels at week 0 in the spelt group, and then decreased with the intervention to levels more similar to the placebo and oat bran groups.

### Individual response analysis

**Figure 7:**
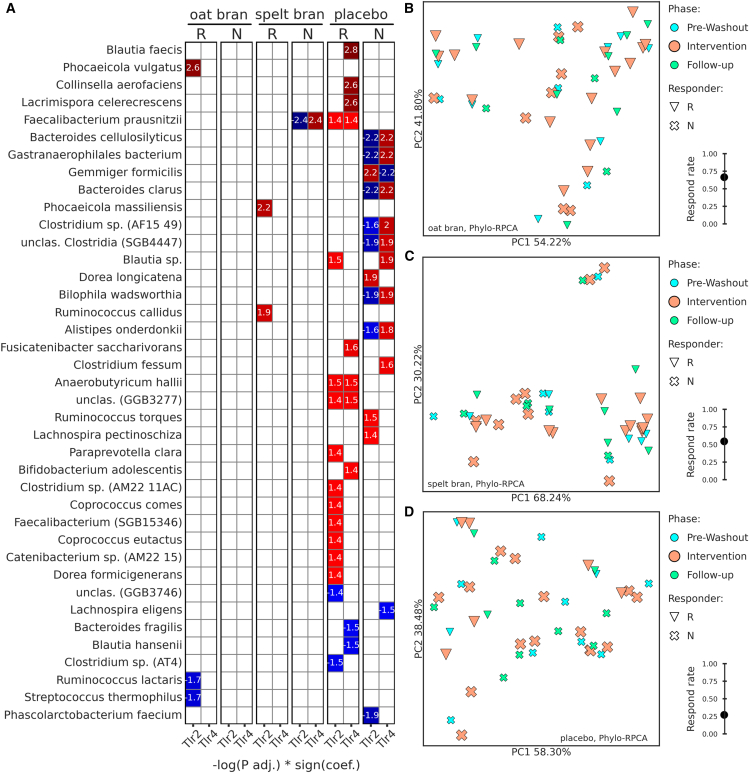
Microbiome composition of responder and non-responder subgroups and correlations with TLR2/TLR4 ligands (A) MaAsLin2 correlations between microbiota abundances and TLR2/TLR4 ligands in responder (R) and non-responder (N) subgroups within each group. (B–D) PcoA plots based on phylo-RPCA distances within each group. Responder (R) and non-responder subgroups are differentiated by markers and phases by color and marker size. In the right lower corner of each PCoA plot, response rate is plotted. Respond rate was calculated as the number of responders divided by the total number of participants within each group.

We hypothesized that changes in microbiota composition might be most pronounced in participants responding to the dietary intervention (defined as a reduction of CAP levels compared to baseline). The oat bran intervention achieved the highest response rate (75%), while spelt bran and placebo groups showed progressively lower response rates (50% and 25%, respectively) (Figures 7B–7D). PCoA analysis did not reveal distinct microbial community structures between responders and non-responders throughout the study phases (Run-in, Intervention, and Follow-up).

Moreover, we aimed to explore the association of TLR2/4 ligands with microbial taxa (Figure 7A). MaAsLin2 correlation analysis identified microbial signatures associated with responder status. We used effect estimation (ee, -log(P-adjusted) ∗ sign(coefficient)) to express the correlation effect and significance (only for taxa with P-adj <0.05). The highest number of significant associations among both responders and non-responders was detected for the placebo group. In the responder’s subgroup, the strongest correlations were detected for *Blautia faecis* (ee = 2.8), *Collinsella aerofaciens* (ee = 2.6) and *Lacrimispora celerecrescens* (ee = 2.6), which were positively associated with TLR4 ligands. Some other species that positively correlated in this subgroup with TLR2/4 (ee = 1.4–1.6), were *Faecalibacterium prausnitzii, Blautia* sp.*, Fusicatenibacter saccharivorans, Anaerobutyricum hallii, Paraprevotella clara, Bifidobacterium adolescentis, and Clostridium* sp. (AM22 11AC), *Coprococcus comes, Coprococcus eutactus and Dorea formicigenerans*. A smaller group of species was found to have negative correlations with TLR2/4 (ee ranging from −1.4 to −1.5): *Bacteroides fragilis, Blautia hansenii,* and *Clostridium* sp. (AT4). Among the participants from the non-responders’ subgroup of the placebo group, some species demonstrated positive correlations with TLR4 (ee = 1.8–2.2) but negative with TLR2 (ee ranging from −1.6 to −2.2). These species were *Bacteroides cellulosilyticus, Gastranaerophilales bacterium, Bacteroides clarus, Clostridium* sp. (AF15 49), *unclassified Clostridia (SGB4447), Bilophila wadsworthia, and Alistipes onderdonkii*. On the contrary, *Gemmiger formicillis* negatively correlated with TLR4 (ee = −2.2) but positively with TLR2 (ee = 2.2) ligands. Other species that positively correlated with TLR2/4 ligands (ee = 1.4–1.9) were *Blautia* sp.*, Dorea longicatena, Clostridium fessum, Ruminococcus torques, and Lachnospira pectinoschiza*. In the same subgroup and conditions, negatively correlated *Lachnospira eligens* (ee = −1.5) and *Phascolarctobacterium faecium* (ee = −1.9). In the oat bran group, significant correlations were only detected among responders, with a positive correlation of *Phocaeicola vulgatus* (ee = 2.6) with TLR2 and a negative correlation of *Ruminococcus lactaris* (ee = −1.7) and *Streptococcus thermophilus* (ee = −1.7). Finally, in the spelt bran group among responders, positive associations were observed for *Phocaeicola massiliensis* (ee = 2.2) and *Ruminococcus callidus* (ee = 1.9). In the same group but among non-responders, *Faecalibacterium prausnitzii* showed a negative correlation with TLR2 (ee = −2.4) and a positive (ee = 2.4) with TLR4.

### Effect size and post-hoc power analyses

Given the reduced sample size (34 completers vs. 66 planned), we calculated effect sizes and post-hoc power for ALT and CAP as primary endpoints. Effect sizes were small (ALT: oat vs. placebo, Cohen’s d = −0.31; spelt vs. placebo, d = −0.14; CAP: oat vs. placebo, d = −0.13; spelt vs. placebo, d = +0.08). For AST, a moderate effect size was observed for oat vs. placebo (d = −0.48). Post-hoc power analyses demonstrated that the achieved sample size provided limited statistical power: 11% for ALT (oat vs. placebo), 6% for ALT (spelt vs. placebo), 6% for CAP (oat vs. placebo), and 5% for CAP (spelt vs. placebo). These findings indicate that the study was underpowered to detect small effects, but the consistently low effect sizes argue against clinically meaningful benefits of fiber supplementation beyond dietary monitoring.

## Discussion

ADLH (“A diet for liver health”), a 20-week, randomized, double-blind, placebo-controlled dietary trial, showed that monitoring dietary intake even in the absence of a structured counseling or caloric restriction resulted in an initial improvement of transaminases and γ-GT and loss of body weight in patients with MASLD. Interestingly and somewhat surprisingly, an addition of dietary fiber supplementation, be it spelt bran or oat bran, did not further improve hepatic steatosis. However, neither the intervention groups nor the placebo group returned to their initial body weight or transaminases and γ-GT, further suggesting that changes in caloric intake and dietary habits introduced by the participants were maintained for more than 20 weeks without any counseling or constant reminding of physicians or nurses. The initial weight loss was associated with an increase in microbiome phylogenetic diversity. After the Run-in phase, neither the introduction of oat bran nor spelt bran consistently changed gut microbiota composition. Moreover, ADLH could not confirm previously reported effects of oat derived β-glucan or oat bran on the serum lipid profile or bile acid composition.^15^^,^^23^

MASLD is the most prevalent liver disease globally and an increasing cause of liver-related morbidity and mortality.^24^ The gut microbiome has been identified as an important factor that can fuel inflammation in MASLD and drive fibrosis progression.^8^^,^^9^ Therefore, therapies that modulate the gut microbiome hold promise to prevent or even reverse liver disease in patients with MASLD.

Dietary measures continue to be fundamental in MASLD care, and the gut microbiome and its metabolites via the gut-liver axis are thought to have a major role in modulating disease severity. Studies in mice suggest that dietary interventions such as time-restricted feeding or intermittent fasting can improve MASLD by enriching intestinal microbiota with beneficial bacteria and metabolites,^25^ and this effect may be mediated by PPARα and PCK1.^26^ However, in humans, most of the highly structured programs where marked caloric restrictions were used were afflicted with rather high drop-out and relapse rates.^27^

Data regarding the effect of oat bran or oat β-glucan on intestinal microbiota are conflicting. In a pilot study in healthy women, while observing a decrease in ALT and AST levels in serum, no effect on gut microbiota was observed.^19^ In contrast, in other studies, the gut microbiome changed following an enrichment of the diet with oat bran or oat β-glucan but also other soluble dietary fibers being related to an enrichment of potentially beneficial taxa, such as *Bacteroides acidifaciens*.^20^^,^^28^^,^^29^

Moreover, oat bran has been explored for its potential to improve the lipid profile and lessen hepatic inflammation. In various animal and clinical studies, the intake of oat bran (oat β-glucan) has been linked to lower low-density lipoprotein cholesterol, reduced hepatic fat deposition, and strengthened intestinal barrier function.^15^^,^^17^ However, in line with the results from this study, our previous preclinical study in a murine model of MASLD, not show improvements in glucose tolerance nor hepatic steatosis after supplementation with oat β-glucan. However, in mice, we observed a pronounced effect on hepatic fibrosis and inflammation, which was linked to the modulation of the gut microbiome and TLR ligands.^20^

Our study has important implications. First, the improvement of liver enzymes and BMI during the Run-in period underlines the importance of detailed nutritional monitoring. The Hawthorne effect, which describes the impact of the monitoring itself without any specific intervention on the participants’ behavior,^30^^,^^31^^,^^32^ may have the potential to impact diet-related behavior of patients with MASLD. However, unbiased dietary data assessments, even with web-based or app-based assessment methods, are still not fully achieved.^33^ Robust Run-in phases and careful dietary assessments are required to differentiate true intervention effects from changes triggered by diet awareness alone. We would like to point out that omitting the Run-in phase would likely have resulted in a different study outcome and false attrition of changes to the dietary intervention. Along the same line, results of dietary studies lacking a Run-in phase and proper nutritional assessment should be interpreted with caution since some previously reported outcomes may reflect heightened participant vigilance about food choices rather than the direct action of a tested dietary intervention. Second, while prior research suggested that oat bran or oat β-glucan might favorably reshape the intestinal microbiota, our data did not reveal marked changes over the relatively short 12-week course. Although minor changes in specific taxa were observed, overall gut microbiota remained largely stable during and after the intervention, possibly due to sample size, study duration, or background dietary habits. The most pronounced differences in microbiota composition were observed between the two study sites, Vienna and Aachen. This is in line with previously reported influences of local dietary and lifestyle habits on gut microbiome composition.^34^^,^^35^

In summary, our study unexpectedly revealed that dietary assessment by itself and in the absence of any counseling and advice by trained personnel can have a more pronounced effect on clinical parameters of MASLD, e.g., transaminases and BMI, than a highly structured, costly dietary intervention. Moreover, robust Run-in phases and careful dietary assessments are advised to differentiate true intervention effects from changes triggered by diet awareness alone. This study underscores the need for enhanced awareness of food intake and dietary habits in patients with metabolic disease. Indeed, dietary counseling and careful assessment of dietary habits should be a key component in the clinical management of MASLD and emphasizing the importance of rigorous controls in dietary studies related to this condition. Our study also suggests that fiber rich dietary supplementations such as oat bran and spelt bran, are well tolerated in individuals with MASLD, bearing no adverse effects, but may not yield additional hepatic or metabolic benefits over fiber poor diets. Further studies are needed to explore the persistence of a dietary assessment, e.g., 3 days of weighing, with respect to improving MASLD and if additional “incentives” or “nudging,” like the consumption of a specific food may enhance adherence to a calorie restricted diet.

### Limitations of the study

Several limitations must be considered when interpreting the data. First, the sample size was limited, which may reduce the power to detect additional changes following the Run-in phase. Second, dropouts, although spread evenly across groups, still reduce power and may influence interpretation. Third, the accuracy of food recording, though carefully performed, still depends on the participant’s compliance and, as seen by the improvement of transaminases and reduction of BMI, may not have reflected the “normal” or “real” intake of patients. Fourth, we noted that local factors (e.g., study center differences) may influence the gut microbiome. Fifth, compliance was only based on self-reported data, while potential biomarkers of dietary fiber intake, such as fecal fiber components or stool weight^36^ haven't been assessed. Moreover, our study was underpowered compared with the initial sample size calculation, mainly due to the COVID-19 pandemic and discontinuation of recruitment at one site. To mitigate this limitation, we performed effect size analyses and post-hoc power calculations. Effect sizes were consistently small (Cohen’s d ≤ 0.31) for ALT and CAP, with corresponding post-hoc power values < 12%. These findings suggest that the absence of significant group differences was not merely attributable to type II error but reflects a true lack of clinically meaningful benefit from fiber supplementation. Importantly, the consistency of null results across liver enzymes, CAP, bile acid profiles, and microbiota composition strengthens our conclusion that dietary monitoring alone accounts for the observed improvements.

Finally, the duration of the intervention may have been insufficient to observe longer-term metabolic shifts attributable to the enrichment of the diet with fiber. Our dietary trial did not include an invasive assessment of steatohepatitis activity via histology. Therefore, our study is also limited in detecting effects on hepatic inflammation and fibrosis.

## Resource availability

### Lead contact

Further information and requests for resources and reagents should be directed to and will be fulfilled by the Lead Contact, Prof. Dr. Kai Markus Schneider (markus.schneider@tu-dresden.de).

### Materials availability

All materials used in this study are available from the sources specified in the table above.

### Data and code availability

• All data and code to understand and assess the conclusions of this research are available in the main text, supplemental information. Metagenomic sequencing data have been deposited at ENA: PRJEB73985 and will be made publicly available. Accession numbers are listed in the key resources table.
• Additionally, raw count tables and code (when available) used in the study were deposited to GitHub (https://github.com/timyerg/Dimiliv/releases/tag/v1.0).
• Any additional information required to reanalyze the data reported in this article is available from the lead contact upon request.

## Acknowledgments

The study has been approved by ethics committees of the two study centers in Germany and Austria, RWTH Aachen (EK 340/17) and Medical University of Vienna (EK Nr 1130/2020).

β-glucan rich oat bran flakes were a gentle gift of Peter Kölln GmbH & Co. The authors acknowledge the support of the High Performance and Cloud Computing Group at the Zentrum für Datenverarbeitung of the University of Tübingen, the state of Baden-Württemberg through bwHPC, and the GermanResearch Foundation (DFG) through grant no. INST 37/935-1FUGG. Graphical Abstract and Figure 1 created with Biorender.com.

This study was supported by HDHL-INTIMIC Di-Mi-Liv to A.C.S., C.T., I.B., K.M.S., and M.T. K.M.S. is supported by the CRC 1382 project B09 funded by Deutsche Forschungsgesellschaft (DFG, 10.13039/501100001659German Research Foundation)—Project-ID 403224013—SFB 1382 as well as 10.13039/501100002347Federal Ministry of Education and Research (BMBF) and the Ministry of Culture and Science of the German State of North Rhine-Westphalia under the Excellence strategy of the federal government. Research was supported by the DRF (2025 – SF – 003) to C.T.

## Author contributions

Author contributions: A.B. and T.Y. conceived the study, designed and performed the experiments, interpreted the results, and wrote the article. E.M., A.C., and J.W.J. recruited patients, performed study visits, and analyzed data. R.G., A.H.A., and C.V.S. performed computational and statistical analyses, W.S. and A.M. performed bile acid analyses, M.T. and C.T. designed the study, interpreted results, provided essential tools, insights, and funding, A.C.S., I.B., and K.M.S. conceived the study, interpreted the results, wrote the article, and provided funding.

## Declaration of interests

The authors declare no competing interests.

## STAR★Methods

### Key resources table

**Table:** 

REAGENT or RESOURCE	SOURCE	IDENTIFIER
**Chemicals, peptides, and recombinant proteins**		

FastDNA™ Spin Kit	MP Biomedicals	116540600-CF

**Critical commercial assays**		

Human TLR2 Reporter HEK293 Cell Assay	Invivogen	Cat# hkb-htlr2, RRID:CVCL_IM80
Human TLR4 Reporter HEK293 Cell Assay	Invivogen	Cat# hkb-htlr4, RRID:CVCL_IM82

**Deposited data**		

Raw data	ENA repository	PRJEB73985
Code	GitHub repository	https://github.com/timyerg/Dimiliv/releases/tag/v1.0

**Software and algorithms**		

GraphPad Prism version 7	GraphPad Software	https://www.graphpad.com
Microsoft Excel	Microsoft	https://www.microsoft.com/en-us/microsoft-365/excel
MetaPhlan4	The Huttenhower lab	https://huttenhower.sph.harvard.edu/metaphlan/
HUMAnN3	The Huttenhower lab	https://huttenhower.sph.harvard.edu/humann/
MaAsLin2	The Huttenhower lab	https://huttenhower.sph.harvard.edu/maaslin/
Qiime2	Qiime2 developing team	https://github.com/qiime2/qiime2
ANCOM-BC	University of Maryland	https://www.bioconductor.org/packages/release/bioc/vignettes/ANCOMBC/inst/doc/ANCOMBC.html
statsmodels Python package (v0.14).	Josef Perktold, Skipper Seabold, Jonathan Taylor, statsmodels-developers.	https://github.com/statsmodels

### Experimental model and study participant details

#### Human participants

The study was initiated at University of Gothenburg (Sweden), University Hospital RWTH Aachen (Germany) and University Hospital for Internal Medicine III, Vienna (Austria). The ethics committees of the three study centers in Germany, Austria, and Sweden, RWTH Aachen and Medical University of Vienna as well as University of Gothenburg approved the present study and the study was carried out in accordance with the ethical standards laid down in the Declaration of Helsinki of 1975 as revised in 1983. Informed consent was obtained from all participants prior to their inclusion in the study. The study was registered at ClinicalTrials.gov (NCT03897218). The sample size calculation taking the three study centers in consideration was based on an effect size of 0.4, a power of 0.8 and an alpha level of 0.05 and resulted in 22 individuals per group. Accordingly, it was planned to enroll 6-8 patients per study center. However, due to serious health issues of the local PI at University of Gothenburg as well as restrictions during the COVID-19 pandemic the recruitment has never been completed at this study site. Thus, the center had to be excluded from the final analyses.

From April 2019 to October 2021, 79 participants (male and female) were assessed for eligibility from the remaining two research centers in Germany and Austria (University Hospital RWTH Aachen and University Hospital for Internal Medicine III, Vienna). Eligible participants were adults (aged 18-80 years) with steatotic liver disease (CAP >280dB) and without fibrosis or evidence of increased alcohol intake (<30g/day for men and <20g/day for women). Further exclusion criteria were any other liver disease (hepatocellular carcinoma, liver cirrhosis (Fibroscan > 12 kPa), hepatitis, Wilson's disease, clinically manifest iron overload, cholestatic liver disease or liver transplantation), treatment with UDCA, vitamin E or different MASH drugs 3 months before randomization, bariatric surgery in the last 5 years, BMI <18.5 kg / m2, - HIV infection, heart failure (New York Heart Association class II - IV), myocardial infarction, unstable coronary artery disease, coronary artery intervention or stroke in the last 6 months, unstable COPD, inflammatory bowel disease or rheumatoid arthritis, unstable renal insufficiency (changes in serum creatinine > 50% in the last 3 months) or terminal renal insufficiency requiring dialysis, uncontrolled hypertension (SBP / DBP> 180/90 despite therapy), uncontrolled metabolic conditions (poorly controlled or decompensated diabetes mellitus, HbA1c >9 %), food allergies or intolerances that require strict adherence to a diet, such as lactose intolerance or coeliac disease, pregnancy or breastfeeding women (medical history), treatment with drugs or substances that can induce secondary MASH (e.g. tamoxifen, corticosteroids, amiodarone, methotrexate) or alleviate MASH (TNF antagonists) (metformin), use of herbal food supplements, antibiotics treatment 6 weeks prior to the study.

#### Study design

The study design of the investigator-initiated, randomized, double-blind, placebo-controlled, dual-centre dietary intervention study conducted at the University Hospital RWTH Aachen in Germany and University Hospital Vienna Medical University of Vienna, University Hospital for Internal Medicine III, in Austria is summarized in Figure 1.

After a three- weeks Run-in phase, during which participants were asked to refrain from consuming foods containing oats as well as pre- and probiotics and all participants were asked to document their dietary intake employing a 3-day weighing protocol, participants were randomly allocated (1:1:1) by block randomization (blocks of 3) to the following three study groups: a group receive daily portions of oat bran, spelt bran, or matched placebo flakes for 12 weeks. The commercially cereal flakes and brans were mixed by the study team to contain either 4.5g oat β-glucan with a total of 11.7 g dietary fiber (oat bran intervention), 11.7 insoluble dietary fiber (spelt bran fiber) or 2.1 g dietary fiber (placebo), (see Table S5) and were matched in calories (∼250kcal/ day). To provide a gradual increase of dietary fiber intake, participants received one third of the flake mixes on day 1 and day 2 and two thirds of the total flake mixes from day 3 to day 5. Participants were asked to consume the flakes in two portions during the day (breakfast, lunch, dinner) and consume them with milk, water, yoghurt or juice. This intervention phase was followed by 8 weeks Follow-up. Dosing and intake recommendations were based on previous trials of our group.^19^

Participants, investigators, and nurses involved in the study were masked to the randomization outcome. The primary endpoint was a decrease in CAP value and ALT-concentration in blood samples from baseline to end of treatment. Exploratory endpoints included fecal microbiota composition and function analyzed using shotgun metagenomics and serum comprehensive bile acid profiling as well as markers of intestinal barrier function in serum.

A total of 48 participants were randomized to receive oat bran (Group A, n = 16), spelt bran (Group B, n = 17) or a placebo (Group C, n = 15) (Figure 1). Of these, 34 completed the entire study protocol, provided all stool samples, and were included in the final analysis (Group A: n = 12; Group B: n = 11; Group C: n = 11). Baseline characteristics are found in Table 2. The sex distribution was equal between groups.

#### Cell lines

Human TLR2/TLR4 Reporter HEK293 Cells were purchased from Invivogen, France and cultured in DMEM medium (PAN-Biotech, Germany) supplemented with 10% fetal bovine serum (FBS), 1% penicillin-streptomycin (PAN-Biotech, Germany), 1% normocin (Invivogen, France) and 1% HEK-Blue Selection (Invivogen, France). Cells were maintained in a humidified incubator at 37°C with 5% CO2, and mycoplasma contamination was routinely tested.

### Method details

#### Sample collection and dietary protocols

Overnight fasted blood samples and fecal samples were collected at timepoints -3W (3 week Run-in), 0 weeks (0W), 2W, 12W and 20W, while also anthropometry was assessed. Fibroscan was performed at -3W, 0W, 12W and 20W. Dietary intake was assessed by 3-day weighing protocol (at least one weekend day) before 0W, 2W, 12W and 20W and EBISpro (Version 2011, Germany) was used to analyze the nutritional data. Nutritional data was also used to assess the Healthy Eating Index (HEI) based on the German National Nutrition Survey II.^37^ Underreporting was assessed as detailed before.^38^ The compliance of the participants was checked and confirmed via the dietary protocols and intervention diaries. In addition, the participants had to return the empty bags of study cereal at each visit. Only compliant participants were included in the analysis.

#### TLR ligands in serum

Ligands for Toll-like receptor (TLR) 2 and 4 have been assessed in serum of participants by employing a commercially available HEK secreted embryonic alkaline phosphatase (SEAP) reporter HEK293 cell assay (Invivogen, France) as detailed before.^39^

#### Bile acid composition in serum

Bile acids were extracted from serum samples via methanol extraction as previously published.^40^ Protein precipitation was achieved by adding 10 volumes of internal standard-containing methanol. The supernatant was evaporated and resuspended in methanol:water (1:1). Bile acids were separated through gradient elution on a Kinetex silica C18 column (2.1 x 100 mm with 1.7 μm particles, Phenomenex, Torrance, CA, USA) at 60 °C. Finally, detection was performed with ultra-performance liquid chromatography tandem mass-spectrometry (UPLC-MS/MS, Wallenberglab laboratory at Sahlgrenska University Hospital, Sweden).

#### DNA extraction and sequencing

DNA from stool was isolated using the DNeasy PowerSoil Pro Kits (Qiagen, Germany) following the manufacturer instructions. After extraction, DNA was quantified using Qubit dsDNA high-sensitivity assay kit (Invitrogen by Thermo Fisher Scientific, USA). Metagenomic sequencing was performed using Illumina Novaseq 6000 NextSeq500 (Illumina) 2 × 150 bp.

### Quantification and statistical analysis

The statistical details of the experiments can also be found in the legend of each respective figure or table. Depending on gaussian distribution, two groups (-3W vs 0W) were compared by paired t-test or Wilcoxon matched-pairs signed rank test while a one-way ANOVA or Kruskal Wallis test was performed when comparing baseline values of three groups (GraphPad Prism (7.03). For between-group comparisons over time (during intervention and follow-up stages) of numerical traits, linear mixed effects analysis^41^ were used with placebo group being treated as the reference.

We calculated Cohen’s d effect sizes for ALT and CAP to quantify the magnitude of between-group differences. Cohen’s d expresses the difference between two group means divided by the pooled standard deviation and provides a standardized, scale-free measure of effect size. Conventionally, d ≈ 0.2 indicates a small, 0.5 a moderate, and ≥0.8 a large effect. To further address the potential risk of type II error, we performed post-hoc power analyses using the statsmodels Python package (v0.14). These analyses were based on the observed effect sizes and group sample sizes, assuming α = 0.05 (two-sided).

For microbiome-related analyses, raw reads were quality-controlled with TrimGalore, which includes Cutadapt^42^ and FastQC tools. Host DNA was removed by Bowtie2 aligner.^43^ Bacterial profiling and functional annotations were performed by MetaPhlan4 and HUMAnN3.^44^ Diversity metrics analyses were performed in Qiime2.^45^ Alpha diversity was assessed by Shannon’s entropy^46^ and Faith’s phylogenetic diversity.^47^ For beta diversity estimation, phylogenetic robust aitchison distances^48^ were calculated. Beta diversity ordination was performed with a principal-coordinate analysis (PCoA).^49^ Alpha diversity metrics were tested with the Wilcoxon test for dependent samples^50^ and the Kruskal-Wallis test^51^ for independent. Statistical analysis of beta diversity distances was performed with Adonis test and PERMANOVA tests.^52^ All P-values obtained from multiple comparisons of diversity metrics were adjusted using the Benjamini-Hochberg procedure.^53^ The differential abundance test at the taxonomy level was carried out by Ancom-BC.^54^ Functional annotations from HUMAnN3 output were tested with MaAsLin2.^55^

### Additional resources

This study was registered at ClinicalTrials.gov under the identifier NCT03897218.
