# Supplementary material for: Fiber enrichment is not superior to dietary monitoring in MASLD: A dual-center, double-blind, placebo-controlled trial
Source: iScience. 2025 Nov 11;28(12):114019. doi: 10.1016/j.isci.2025.114019 (PMC12721201; doi:10.1016/j.isci.2025.114019)
Supplement: Document S1. Figures S1–S9 [file mmc1.pdf]

iScience, Volume *28*

Supplemental information

Fiber enrichment is not superior to dietary monitoring in MASLD: A dual-center, double-blind, placebo-controlled trial

Annette Brandt, Timur Yergaliyev, Emina Halibasic, Aline Cyba, Julius W. Jaeger, Rongpeng Gong, Angélica Hernández-Arriaga, Carolin Victoria Schneider, Wilhelm Sjöland, Antonio Molinaro, Michael Trauner, Christian Trautwein, Amélia Camarinha-Silva, Ina Bergheim, and Kai Markus Schneider

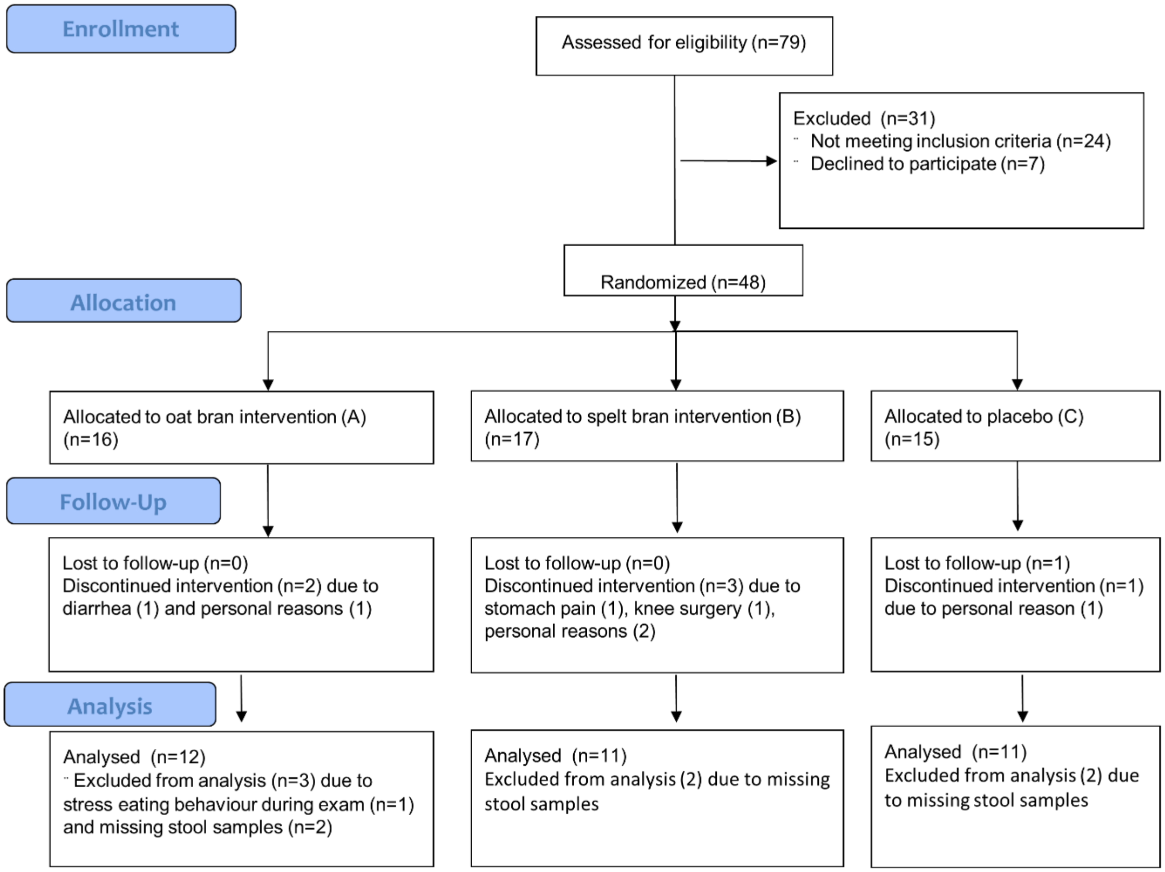

Figure S1: CONSORT Flow Diagram

Table S1: Adonis test output by phase.

Factor	R2	P-value	Mode	Group1	Group2	P-adj.	Phase
Group	0.079	0.216	General	-	-	-	Run-in
Sex	0.026	0.349		-	-	-	
Location	0.152	0.006		-	-	-	
Group	0.069	0.019		-	-	-	Intervention
Sex	0.03	0.071		-	-	-	
Location	0.207	0.001		-	-	-	
Group	-	-	Pairwise	B	C	0.837	
	-	-		B	A	0.042	
	-	-		C	A	0.147	
Group	0.065	0.258	General	-	-	-	Follow-up
Sex	0.006	0.77		-	-	-	
Location	0.221	0.001		-	-	-	

Table S2: Full model statistics of Linear mixed effects (LME) analyses of Table 3

Parameter	Coef.	Std.Err.	z	P>|z|	[0.025	0.975]	Factor	Period	Converged
Group[T.Oat]	47,767	260,333	0,183	0,854	-462,476	558,01	kcal	Intervention	Yes
Group[T.Spelt]	-430,211	260,333	-1,653	0,098	-940,454	80,031	kcal	Intervention	Yes
Week	-8,011	13,521	-0,592	0,554	-34,512	18,49	kcal	Intervention	Yes
Week:Group[T.Oat]	-4,656	19,793	-0,235	0,814	-43,449	34,137	kcal	Intervention	Yes
Week:Group[T.Spelt]	22,266	19,793	1,125	0,261	-16,528	61,059	kcal	Intervention	Yes
Group[T.Oat]	8,275	10,216	0,81	0,418	-11,749	28,299	g protein	Intervention	Yes
Group[T.Spelt]	-15,926	10,216	-1,559	0,119	-35,95	4,097	g protein	Intervention	Yes
Week	0,096	0,671	0,143	0,887	-1,22	1,411	g protein	Intervention	Yes
Week:Group[T.Oat]	-1,117	0,983	-1,137	0,256	-3,043	0,809	g protein	Intervention	Yes
Week:Group[T.Spelt]	0,651	0,983	0,663	0,507	-1,274	2,577	g protein	Intervention	Yes
Group[T.Oat]	4,215	3,39	1,243	0,214	-2,429	10,859	g dietary fiber	Intervention	Yes
Group[T.Spelt]	2,054	3,39	0,606	0,545	-4,59	8,698	g dietary fiber	Intervention	Yes
Week	-0,14	0,206	-0,68	0,497	-0,544	0,264	g dietary fiber	Intervention	Yes
Week:Group[T.Oat]	0,448	0,302	1,487	0,137	-0,143	1,039	g dietary fiber	Intervention	Yes
Week:Group[T.Spelt]	0,79	0,302	2,621	0,009	0,199	1,381	g dietary fiber	Intervention	Yes
Group[T.Oat]	7,775	14,031	0,554	0,579	-19,725	35,275	g fat	Intervention	Yes
Group[T.Spelt]	-9,341	14,031	-0,666	0,506	-36,841	18,159	g fat	Intervention	Yes
Week	-0,303	0,859	-0,353	0,724	-1,986	1,38	g fat	Intervention	Yes
Week:Group[T.Oat]	-0,861	1,257	-0,685	0,493	-3,325	1,602	g fat	Intervention	Yes
Week:Group[T.Spelt]	0,482	1,257	0,383	0,702	-1,982	2,945	g fat	Intervention	Yes
Group[T.Oat]	-3,878	34,928	-0,111	0,912	-72,335	64,58	g carbohydrates	Intervention	Yes
Group[T.Spelt]	-49,614	34,928	-1,42	0,155	-118,072	18,843	g carbohydrates	Intervention	Yes
Week	0,448	1,529	0,293	0,77	-2,548	3,444	g carbohydrates	Intervention	Yes
Week:Group[T.Oat]	0,552	2,238	0,247	0,805	-3,834	4,938	g carbohydrates	Intervention	Yes

Week:Group[T.Spelt]	1,593	2,238	0,712	0,476	-2,793	5,979	g carbohydrates	Intervention	Yes
Group[T.Oat]	2,146	4,066	0,528	0,598	-5,824	10,116	HEI	Intervention	Yes
Group[T.Spelt]	3,499	4,066	0,86	0,39	-4,471	11,469	HEI	Intervention	Yes
Week	-0,288	0,268	-1,073	0,283	-0,814	0,238	HEI	Intervention	Yes
Week:Group[T.Oat]	0,136	0,393	0,347	0,729	-0,634	0,907	HEI	Intervention	Yes
Week:Group[T.Spelt]	-0,004	0,393	-0,009	0,992	-0,774	0,767	HEI	Intervention	Yes
Group[T.Oat]	-50,688	535,277	-0,095	0,925	-1099,813	998,436	kcal	Follow-up	Yes
Group[T.Spelt]	-155,967	535,277	-0,291	0,771	-1205,091	893,157	kcal	Follow-up	Yes
Week	-11,458	19,275	-0,594	0,552	-49,236	26,32	kcal	Follow-up	Yes
Week:Group[T.Oat]	4,592	28,216	0,163	0,871	-50,71	59,893	kcal	Follow-up	Yes
Week:Group[T.Spelt]	-1,096	28,216	-0,039	0,969	-56,397	54,206	kcal	Follow-up	Yes
Group[T.Oat]	-27,683	18,5	-1,496	0,135	-63,941	8,575	g protein	Follow-up	Yes
Group[T.Spelt]	-15,576	18,5	-0,842	0,4	-51,834	20,683	g protein	Follow-up	Yes
Week	-1,733	0,635	-2,728	0,006	-2,978	-0,488	g protein	Follow-up	Yes
Week:Group[T.Oat]	1,958	0,93	2,106	0,035	0,135	3,78	g protein	Follow-up	Yes
Week:Group[T.Spelt]	0,681	0,93	0,732	0,464	-1,141	2,503	g protein	Follow-up	Yes
Group[T.Oat]	20,853	6,021	3,463	0,001	9,051	32,654	g dietary fiber	Follow-up	Yes
Group[T.Spelt]	21,324	6,021	3,542	0	9,523	33,125	g dietary fiber	Follow-up	Yes
Week	-0,073	0,199	-0,369	0,712	-0,463	0,316	g dietary fiber	Follow-up	Yes
Week:Group[T.Oat]	-0,991	0,291	-3,406	0,001	-1,561	-0,421	g dietary fiber	Follow-up	Yes
Week:Group[T.Spelt]	-0,869	0,291	-2,988	0,003	-1,44	-0,299	g dietary fiber	Follow-up	Yes
Group[T.Oat]	-10,254	29,76	-0,345	0,73	-68,582	48,075	g fat	Follow-up	Yes
Group[T.Spelt]	-1,989	29,76	-0,067	0,947	-60,318	56,339	g fat	Follow-up	Yes
Week	0,616	1,141	0,54	0,59	-1,621	2,852	g fat	Follow-up	Yes
Week:Group[T.Oat]	0,69	1,67	0,413	0,68	-2,584	3,963	g fat	Follow-up	Yes
Week:Group[T.Spelt]	-0,162	1,67	-0,097	0,923	-3,436	3,112	g fat	Follow-up	Yes

Group[T.Oat]	24,34	71,292	0,341	0,733	-115,389	164,069	g carbohydrates	Follow-up	Yes
Group[T.Spelt]	-21,867	71,292	-0,307	0,759	-161,596	117,862	g carbohydrates	Follow-up	Yes
Week	-2,22	2,603	-0,853	0,394	-7,322	2,882	g carbohydrates	Follow-up	Yes
Week:Group[T.Oat]	-1,519	3,811	-0,399	0,69	-8,988	5,95	g carbohydrates	Follow-up	Yes
Week:Group[T.Spelt]	-0,606	3,811	-0,159	0,874	-8,075	6,862	g carbohydrates	Follow-up	Yes
Group[T.Oat]	19,837	14,243	1,393	0,164	-8,079	47,753	HEI	Follow-up	Yes
Group[T.Spelt]	6,294	14,243	0,442	0,659	-21,622	34,21	HEI	Follow-up	Yes
Week	0,073	0,571	0,129	0,898	-1,046	1,193	HEI	Follow-up	Yes
Week:Group[T.Oat]	-1,354	0,836	-1,62	0,105	-2,992	0,284	HEI	Follow-up	Yes
Week:Group[T.Spelt]	-0,254	0,836	-0,304	0,761	-1,892	1,384	HEI	Follow-up	Yes

Table S3: Full model statistics of Linear mixed effects (LME) analyses of Table 4

Parameter	Coef.	Std.Err.	z	P>|z|	[0.025	0.975]	Factor	Period
Group[T.Oat]	0,465	8,079	0,058	0,954	-15,37	16,301	BMI	Intervention
Group[T.Spelt]	0,004	8,329	0	1	-16,321	16,329	BMI	Intervention
Week	0,042	0,831	0,05	0,96	-1,587	1,67	BMI	Intervention
Week:Group[T.Oat]	-0,008	1,15	-0,007	0,995	-2,262	2,247	BMI	Intervention
Week:Group[T.Spelt]	-0,064	1,177	-0,054	0,957	-2,372	2,244	BMI	Intervention
Group[T.Oat]	-15,781	26,839	-0,588	0,557	-68,385	36,822	Triglyceride	Intervention
Group[T.Spelt]	25,597	27,255	0,939	0,348	-27,822	79,016	Triglyceride	Intervention
Week	0,878	2,269	0,387	0,699	-3,569	5,326	Triglyceride	Intervention
Week:Group[T.Oat]	0,725	3,131	0,232	0,817	-5,412	6,862	Triglyceride	Intervention
Week:Group[T.Spelt]	0,987	3,192	0,309	0,757	-5,27	7,244	Triglyceride	Intervention
Group[T.Oat]	-0,434	15,014	-0,029	0,977	-29,861	28,993	LiverStiffness	Intervention
Group[T.Spelt]	-0,136	15,337	-0,009	0,993	-30,196	29,924	LiverStiffness	Intervention
Week	-0,016	1,278	-0,013	0,99	-2,521	2,489	LiverStiffness	Intervention
Week:Group[T.Oat]	0,619	1,769	0,35	0,726	-2,848	4,087	LiverStiffness	Intervention
Week:Group[T.Spelt]	-0,157	1,807	-0,087	0,931	-3,7	3,385	LiverStiffness	Intervention
Group[T.Oat]	-12,223	13,739	-0,89	0,374	-39,151	14,704	Cholesterol	Intervention
Group[T.Spelt]	6,631	13,916	0,477	0,634	-20,643	33,905	Cholesterol	Intervention
Week	1,143	1,383	0,827	0,408	-1,568	3,854	Cholesterol	Intervention
Week:Group[T.Oat]	-1,105	1,934	-0,571	0,568	-4,895	2,685	Cholesterol	Intervention
Week:Group[T.Spelt]	-0,943	1,946	-0,485	0,628	-4,757	2,871	Cholesterol	Intervention
Group[T.Oat]	7,221	12,028	0,6	0,548	-16,352	30,795	GGT	Intervention
Group[T.Spelt]	12,819	12,215	1,05	0,294	-11,121	36,76	GGT	Intervention
Week	0,564	0,955	0,591	0,555	-1,308	2,436	GGT	Intervention
Week:Group[T.Oat]	-0,257	1,325	-0,194	0,846	-2,855	2,34	GGT	Intervention

Week:Group[T.Spelt]	0,065	1,351	0,048	0,962	-2,583	2,712	GGT	Intervention
Group[T.Oat]	1,107	30,669	0,036	0,971	-59,004	61,218	BMI	Follow-up
Group[T.Spelt]	-1,874	31,329	-0,06	0,952	-63,278	59,53	BMI	Follow-up
Week	-0,054	1,343	-0,04	0,968	-2,687	2,578	BMI	Follow-up
Week:Group[T.Oat]	-0,064	1,86	-0,035	0,972	-3,709	3,58	BMI	Follow-up
Week:Group[T.Spelt]	0,09	1,9	0,047	0,962	-3,633	3,813	BMI	Follow-up
Group[T.Oat]	77,426	86,67	0,893	0,372	-92,444	247,296	Triglyceride	Follow-up
Group[T.Spelt]	185,478	88,534	2,095	0,036	11,955	359,002	Triglyceride	Follow-up
Week	4,43	3,675	1,206	0,228	-2,772	11,633	Triglyceride	Follow-up
Week:Group[T.Oat]	-7,038	5,088	-1,383	0,167	-17,01	2,933	Triglyceride	Follow-up
Week:Group[T.Spelt]	-11,946	5,197	-2,299	0,022	-22,132	-1,759	Triglyceride	Follow-up
Group[T.Oat]	-11,698	51,403	-0,228	0,82	-112,445	89,049	LiverStiffness	Follow-up
Group[T.Spelt]	-12,552	52,508	-0,239	0,811	-115,466	90,362	LiverStiffness	Follow-up
Week	-0,136	2,251	-0,06	0,952	-4,549	4,276	LiverStiffness	Follow-up
Week:Group[T.Oat]	1,558	3,117	0,5	0,617	-4,551	7,667	LiverStiffness	Follow-up
Week:Group[T.Spelt]	0,877	3,184	0,276	0,783	-5,363	7,117	LiverStiffness	Follow-up
Group[T.Oat]	-38,11	52,533	-0,725	0,468	-141,073	64,854	Cholesterol	Follow-up
Group[T.Spelt]	-11,32	52,811	-0,214	0,83	-114,827	92,187	Cholesterol	Follow-up
Week	-1,006	2,264	-0,444	0,657	-5,444	3,432	Cholesterol	Follow-up
Week:Group[T.Oat]	0,973	3,169	0,307	0,759	-5,238	7,185	Cholesterol	Follow-up
Week:Group[T.Spelt]	0,491	3,202	0,153	0,878	-5,785	6,767	Cholesterol	Follow-up
Group[T.Oat]	27,689	40,682	0,681	0,496	-52,046	107,424	GGT	Follow-up
Group[T.Spelt]	5,603	41,557	0,135	0,893	-75,848	87,053	GGT	Follow-up
Week	0,222	1,774	0,125	0,901	-3,256	3,7	GGT	Follow-up
Week:Group[T.Oat]	-2,064	2,457	-0,84	0,401	-6,879	2,751	GGT	Follow-up
Week:Group[T.Spelt]	0,618	2,51	0,246	0,806	-4,301	5,536	GGT	Follow-up

Table S4: Serum bile acid composition.

Bile acids	oat bran				spelt bran				placebo			
	0W	2W	12W	20W	0W	2W	12W	20W	0W	2W	12W	20W
UDCA (nmol/L)	43.37 [21.68;122.45]	71.43 [45.92;161.35]	79.08 [24.87;102.68]	82.91 [43.37;186.22]	79.08 [29.34;168.37]	30.61 [16.58;102.04]	56.12 [21.68;96.94]	14.03 [10.20;31.25]	30.61 [16.58;76.53]	30.61 [7.65;71.43]	42.09 [12.76;125.00]	35.71 [21.68;52.30]
iso-UDCA (nmol/L)	288.27 [43.37;427.30]	279.34 [62.50;432.40]	172.19 [79.72;460.46]	264.03 [80.36;387.76]	242.35 [131.38;596.94]	140.31 [125.00;349.49]	352.04 [168.37;423.47]	190.05 [89.92;311.22]	117.35 [99.49;487.24]	102.04 [61.22;466.84]	312.50 [49.11;524.23]	96.94 [68.88;285.71]
CDCA (nmol/L)	132.65 [67.60;251.28]	174.74 [117.35;358.42]	159.44 [68.88;190.69]	179.85 [104.59;280.61]	331.63 [141.58;562.50]	145.41 [29.34;302.30]	117.35 [48.47;401.79]	80.36 [29.34;156.25]	66.33 [44.64;190.05]	76.53 [43.37;193.88]	100.77 [63.14;174.74]	96.94 [79.08;184.95]
DCA (nmol/L)	278.06 [179.85;489.80]	341.84 [205.99;646.05]	332.91 [199.62;503.19]	348.21 [239.16;535.71]	267.86 [147.96;468.11]	265.31 [158.16;312.50]	242.35 [158.16;488.52]	186.22 [98.85;354.59]	193.88 [80.36;377.55]	165.82 [33.16;181.12]	250.00 [130.10;506.38]	280.61 [98.21;808.67]
HDCA (nmol/L)	43.37 [24.23;61.22]	51.02 [18.49;77.17]	44.64 [21.68;56.76]	36.99 [17.22;44.01]	22.96 [0.00;33.16]	15.31 [1.28;35.71]	22.96 [5.10;31.89]	11.48 [0.64;24.23]	30.61 [14.03;47.19]	22.96 [0.00;33.16]	30.61 [21.68;49.74]	45.92 [17.86;96.94]
CA (nmol/L)	39.22 [23.28;50.25]	183.82 [26.35;337.62]	51.47 [35.54;79.66]	57.60 [19.00;129.90]	132.35 [77.21;817.40]	29.41 [18.38;575.98]	95.59 [33.09;346.81]	36.76 [11.64;120.71]	36.76 [17.16;53.92]	49.02 [17.16;75.98]	40.44 [15.93;56.99]	51.47 [23.28;75.98]
HCA (nmol/L)	2.45 [1.23;7.35]	6.13 [4.29;9.80]	4.90 [2.45;7.97]	4.90 [1.84;15.32]	12.25 [7.35;17.16]	2.45 [1.23;9.80]	2.45 [2.45;8.58]	2.45 [0.61;8.58]	2.45 [0.00;6.13]	2.45 [0.00;4.90]	6.13 [0.00;7.35]	4.90 [1.23;7.35]
LCA (nmol/L)	18.62 [11.97;21.28]	15.96 [9.31;29.92]	17.29 [10.64;23.94]	9.31 [5.32;15.96]	10.64 [2.66;15.96]	13.30 [5.32;18.62]	10.64 [5.32;21.28]	11.97 [7.98;17.95]	7.98 [3.99;22.61]	7.98 [2.66;13.30]	13.30 [9.31;17.29]	13.30 [7.98;30.59]
G-UDCA (nmol/L)	113.59 [23.39;165.92]	108.02 [49.55;164.81]	109.13 [45.10;213.81]	110.24 [72.38;207.68]	153.67 [41.20;212.69]	55.68 [36.75;140.31]	144.77 [65.70;325.17]	63.47 [29.51;91.87]	80.18 [35.63;167.04]	66.82 [24.50;146.99]	47.88 [24.50;182.63]	75.72 [32.29;174.83]
G-CDCA (nmol/L)	614.70 [256.12;1042.32]	545.66 [493.88;1287.31]	714.92 [514.48;1782.85]	804.01 [394.77;1037.86]	650.33 [273.94;962.14]	654.79 [396.44;1105.79]	1024.50 [453.23;1331.85]	600.22 [406.46;889.20]	579.06 [325.17;1177.06]	463.25 [440.98;565.70]	586.86 [327.39;1291.20]	866.37 [340.76;1621.38]
G-DCA (nmol/L)	233.85 [111.36;595.77]	249.44 [144.21;801.78]	246.10 [166.48;775.06]	286.19 [99.67;399.22]	120.27 [73.50;447.66]	129.18 [81.29;316.26]	195.99 [162.58;622.49]	183.74 [84.63;267.82]	207.13 [84.63;438.75]	160.36 [55.68;227.17]	269.49 [108.02;582.41]	387.53 [209.35;659.24]
G-CA (nmol/L)	144.09 [69.89;312.90]	225.81 [84.95;597.31]	194.62 [119.89;367.20]	159.14 [105.38;334.41]	193.55 [88.17;336.56]	200.00 [161.29;303.23]	200.00 [122.58;361.29]	134.41 [98.39;200.54]	144.09 [72.04;211.83]	126.88 [94.62;148.39]	119.35 [76.34;220.43]	200.00 [121.51;389.25]
G-LCA (nmol/L)	13.86 [6.93;35.80]	12.70 [6.35;40.42]	16.17 [12.70;20.79]	13.86 [4.62;19.05]	6.93 [2.31;15.01]	6.93 [3.46;15.01]	13.86 [5.77;19.63]	6.93 [4.62;11.55]	6.93 [4.62;17.32]	6.93 [2.31;11.55]	8.08 [4.62;30.60]	16.17 [3.46;54.27]
T-UDCA	6.01 [2.00;18.04]	4.01 [2.00;9.52]	2.00 [0.00;14.53]	7.01 [2.00;12.53]	8.02 [1.00;18.04]	4.01 [1.00;11.02]	8.02 [2.00;24.05]	4.01 [0.00;5.51]	4.01 [2.00;7.01]	2.00 [0.00;8.02]	2.00 [2.00;14.53]	4.01 [3.01;12.02]

**(nmol/L)**												
**T-CDCA (nmol/L)**	162.32 [43.09;337.68]	110.22 [54.61;260.02]	120.24 [45.59;214.93]	116.23 [67.64;288.08]	140.28 [52.10;298.60]	236.47 [110.22;287.58]	174.35 [84.17;339.68]	130.26 [49.60;206.91]	84.17 [65.13;246.49]	92.18 [30.06;162.32]	133.27 [49.60;219.44]	114.23 [72.14;300.60]
**T-DCA (nmol/L)**	58.12 [29.06;288.58]	50.10 [28.06;239.98]	77.15 [24.05;129.76]	66.13 [22.04;155.31]	44.09 [18.04;93.19]	82.16 [25.05;133.27]	150.30 [39.08;180.36]	65.13 [13.53;109.72]	42.08 [25.05;206.41]	36.07 [20.04;106.21]	90.18 [37.07;192.89]	94.19 [42.08;326.65]
**T-CA (nmol/L)**	31.07 [12.62;74.76]	40.78 [15.05;94.66]	33.98 [9.22;50.49]	26.21 [15.05;69.90]	23.30 [15.53;67.96]	44.66 [28.16;61.17]	36.89 [21.36;61.17]	22.33 [21.36;34.95]	17.48 [11.65;43.69]	19.42 [13.59;33.01]	30.10 [10.68;38.83]	29.13 [18.45;57.28]
**T-LCA (nmol/L)**	2.00 [0.00;6.01]	0.00 [0.00;4.01]	1.00 [0.00;2.51]	0.00 [0.00;0.50]	0.00 [0.00;0.00]	0.00 [0.00;2.00]	2.00 [0.00;3.01]	0.00 [0.00;2.00]	0.00 [0.00;3.01]	0.00 [0.00;0.00]	2.00 [0.00;5.01]	2.00 [0.00;15.03]
**Total BA [nM]**	2715.15 [1736.49;4347.77]	3032.56 [2001.84;4993.24]	3072.71 [1804.53;4715.64]	3347.75 [2122.71;4888.53]	3916.52 [2644.21;4280.55]	2419.78 [2228.30;3941.21]	3466.84 [2309.31;4618.71]	2017.68 [1893.43;3127.05]	3204.01 [1270.49;3800.06]	1848.66 [1140.01;2993.04]	2145.78 [1792.29;4124.26]	3226.31 [2114.66;5336.72]
**C4(ng/mL)**	26.00 [9.97;29.50]	36.00 [25.73;51.46]	35.99 [17.25;41.05]	19.50 [13.75;27.73]	24.00 [8.89;40.71]	26.00 [16.13;48.00]	28.00 [16.43;43.30]	25.18 [16.12;41.50]	33.50 [19.50;48.71]	33.25 [19.34;52.00]	31.24 [25.80;36.49]	22.00 [13.74;25.25]
**C4nmol/L**	64.90 [24.90;73.63]	89.85 [64.23;128.44]	89.83 [43.06;102.46]	48.67 [34.32;69.22]	59.90 [22.19;101.61]	64.90 [40.26;119.82]	69.89 [41.01;108.06]	62.84 [40.22;103.58]	83.61 [48.67;121.58]	82.99 [48.27;129.79]	77.97 [64.40;91.08]	54.91 [34.29;63.02]

Median [Q1;Q3]. oat bran n=12, except for 0W n=11, spelt bran n=11, except for 20W n=10, placebo n=11, except for 2W n=9. Kruskal-Wallis test for overall group differences and pairwise Wilcoxon rank sum tests with Benjamini-Hochberg correction for multiple comparisons.

Table S5: Composition of dietary interventions.

	oat bran	spelt bran	placebo
Oat bran flakes	60 g		
Millet porridge	10 g	10 g	64 g
Spelt bran		15 g	
Semolina cereal		47 g	
Energy/ portion	249 kcal	251 kcal	250 kcal
Dietary fiber/ portion	11.7 g (of which 4.5 g oat β-glucan)	11.7 g	2.1 g
